# Supplementary material for: Alcohol exposure disrupts mu opioid receptor-mediated long-term depression at insular cortex inputs to dorsolateral striatum
Source: Nat Commun. 2018 Apr 3;9:1318. doi: 10.1038/s41467-018-03683-1 (PMC5882774; doi:10.1038/s41467-018-03683-1)
Supplement: Supplementary file 1 — Supplementary Information(PDF 1243 kb) [file 41467_2018_3683_MOESM1_ESM.pdf]

Title: Alcohol exposure disrupts mu opioid receptor-mediated long-term depression at insular cortex inputs to dorsolateral striatum

Munoz et al.

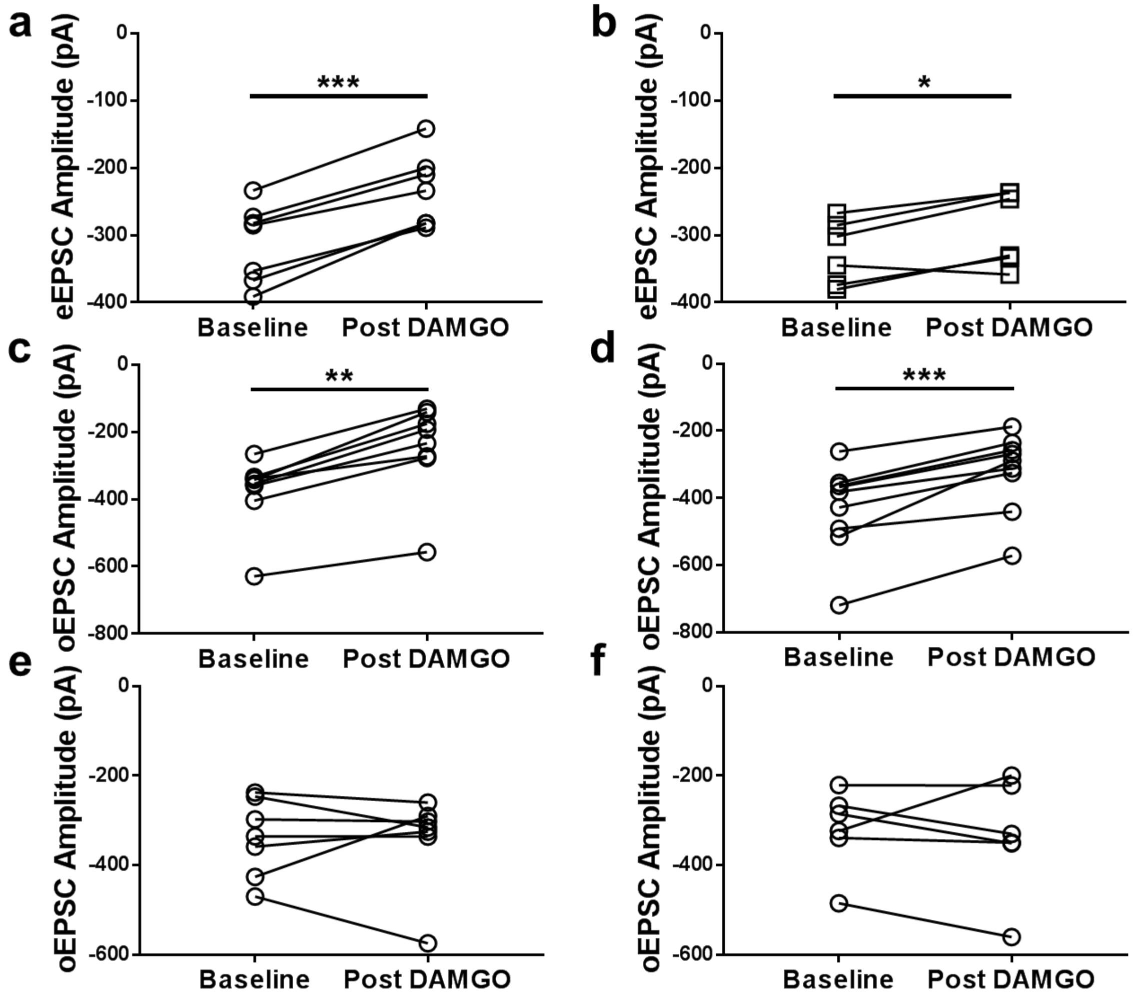

Supplementary Figure 1. MOR activation by DAMGO leads to a reduction of EPSC amplitude. a) DAMGO application (0.3 μM, 5 min) reduced eEPSC amplitude in the DLS, confirming that the activation of MORs leads to mOP-LTD of glutamate release (paired t-test, P=0.0001, t_6=10.88, n=7 from 2 mice). **b)** Reduction of eEPSC amplitude post-DAMGO application confirmed the presence of mOP-LTD in the DMS (paired t-test, P=0.0196, t_5=3.386, n=6 from 2 mice). **c)** DAMGO application (0.3 μM, 5 min) reduced oEPSC amplitude from cortical inputs in the DLS, confirming that the activation of MORs from cortical synapses leads to mOP-LTD (Wilcoxon matched-pairs signed rank test, P=0.0078, n=8 from 7 mice). **d)** Reduction of oEPSC amplitude post-DAMGO application in Ai32-Emx1Cre+ mice confirmed the presence of mOP-LTD mediated by the activation of cortical inputs in the DMS (paired t-test, P=0.0002, t_8=3.386, n=9 from 5 mice). **e)** MOR activation from thalamic inputs in Ai32-Vglut2Cre+ mice does not produce LTD in the DLS (Wilcoxon matched-pairs signed rank test, P=0.6875, n=7 from 3 mice). **f)** DAMGO application does not produce LTD in the DMS (paired t-test, P=0.64, t_5=0.4974, n=6 from 2 mice). *P < 0.05, **P< 0.01, ***P < 0.001.

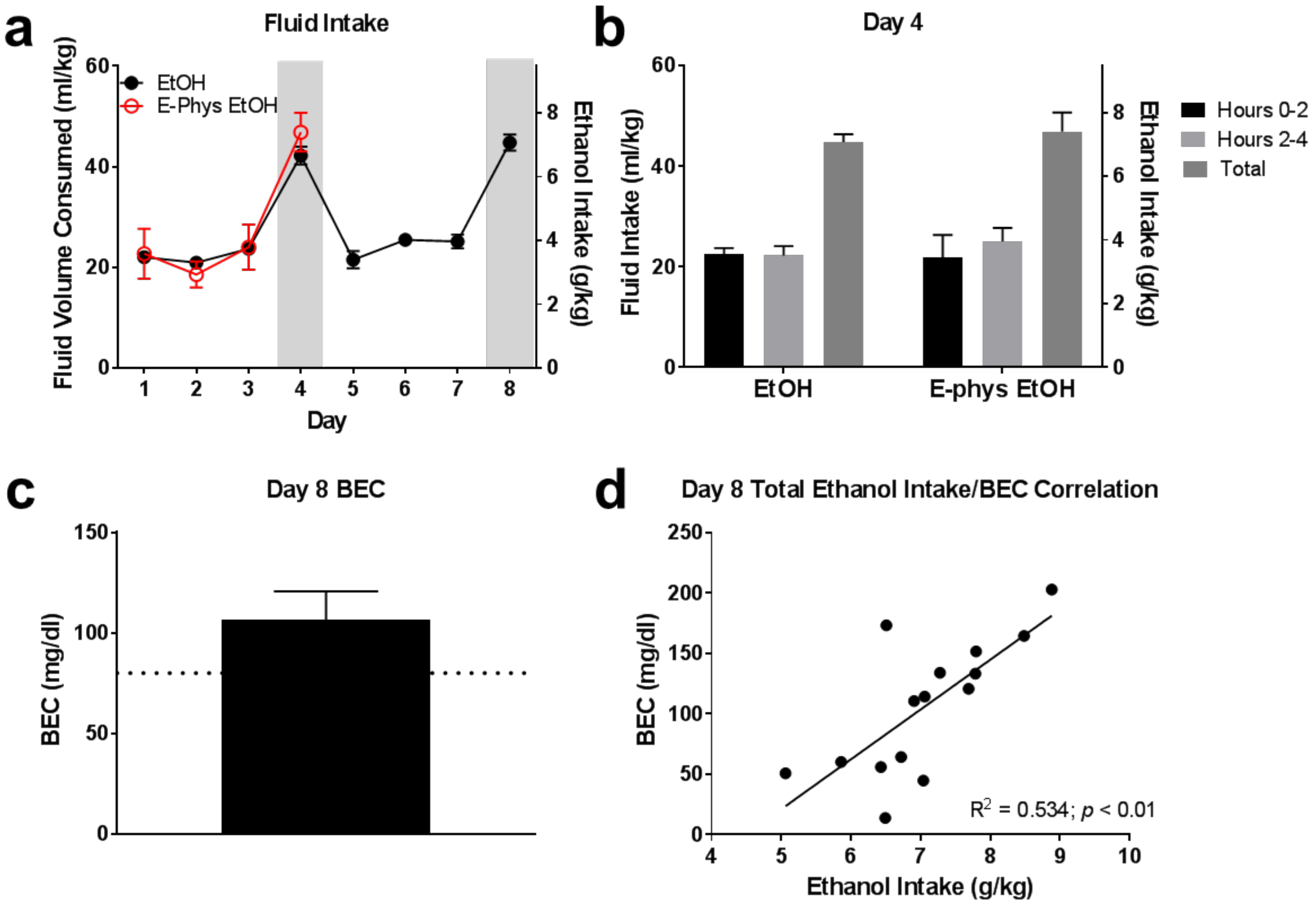

Supplementary Figure 2. EtOH consumption in the DID paradigm. a) Consumption of 20% (v/v) EtOH by the additional cohort of mice as well as mice used in electrophysiology experiments during the 2-4 hour drinking sessions in DID. The gray shaded bars represent 4 hr sessions rather than the 2 hr sessions on the other days. **b)** Electrophysiology mice and the parallel cohort demonstrated equivalent EtOH intake during the 4 hr, day 4 DID session. **c)** Average blood ethanol concentration (BEC) of the parallel cohort mice following the day 8 DID session. **d)** EtOH intake on day 8 was significantly predictive of BEC in the parallel cohort of mice (R^2 = 0.534, P < 0.01, linear regression). *n* = 4 for E-phys EtOH group and 15 for EtOH group. Data represent mean ± SEM.

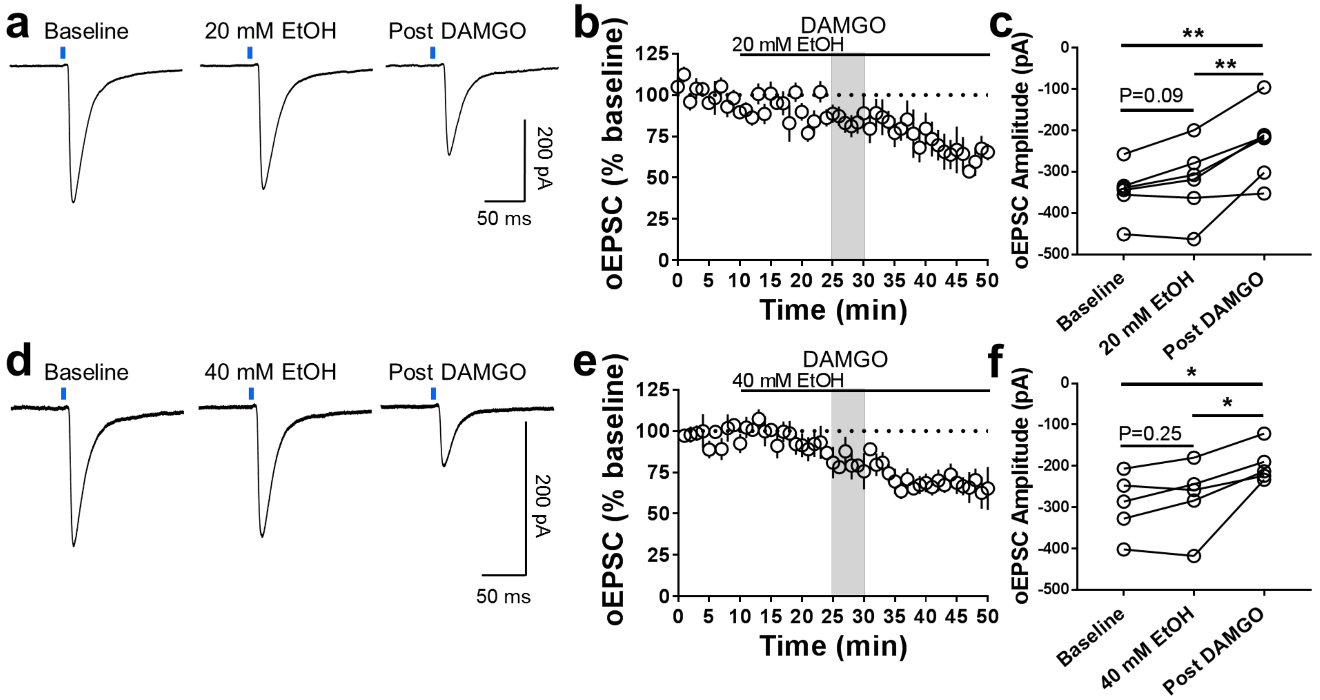

Supplementary Figure 3. EtOH bath application does not induce or block mOP-LTD. **a)** Representative electrically evoked synaptic traces at baseline, during EtOH application (20 mM, 15 min), and after DAMGO application (0.3 µM, 5 min) in combination with EtOH. **b-c)** EtOH does not induce glutamatergic synaptic depression and was incapable of preventing mOP-LTD via DAMGO application (Baseline v. EtOH: P=0.0935, t_5=2.068; Baseline v. DAMGO: P=0.0044, t_5=4.92; EtOH v. DAMGO: P=0.0073, t_5=4.364, n=6 from 2 mice). **d)** Representative electrically evoked synaptic traces at baseline, during EtOH application (40 mM, 15 min), and after DAMGO application (0.3 µM, 5 min) in combination with EtOH. **e-f)** Similar to the 20 mM concentration, 40 mM EtOH does not induce glutamatergic synaptic depression and was incapable of preventing mOP-LTD via DAMGO application (Baseline v. EtOH: P=0.251, t_4=1.341; Baseline v. DAMGO: P=0.0139, t_4=4.183; EtOH v. DAMGO: P=0.0388, t_4=3.029, n=5 from 2 mice). Data represent mean ± SEM. *P < 0.05, **P < 0.01.

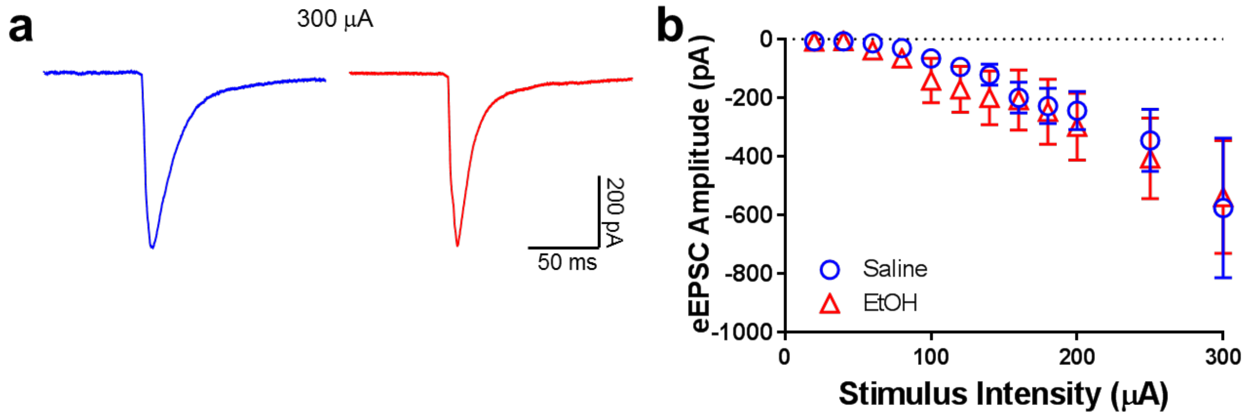

Supplementary Figure 4. Stimulus-response intensity in DLS MSNs was not affected by *in vivo* EtOH exposure. a) Representative electrically evoked synaptic traces of DLS MSNs from C57BL/6J mice injected with saline (blue circles) or 2.0 g/kg EtOH (red triangles) 24 h earlier. **b)** EtOH does not affect the amplitude of eEPSCs in the DLS (2-way repeated measures ANOVA with Sidak's multiple comparisons test, $F(1, 11) = 0.154$, $p=0.7022$, saline v. EtOH, n =7 EtOH and n= 6 saline from 1 mouse each). Data represent mean ± SEM.

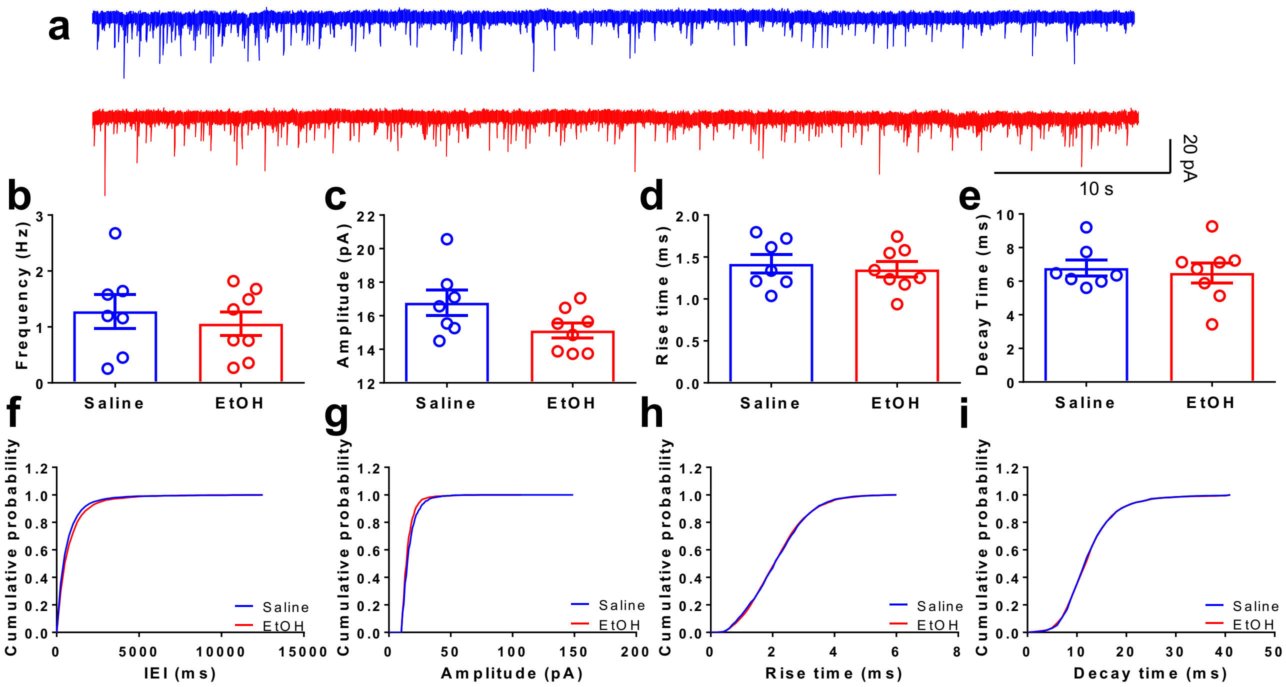

Supplementary Figure 5. A single *in vivo* EtOH exposure does not affect DLS MSN sEPSCs. a) Representative spontaneous excitatory postsynaptic current (sEPSC) traces of saline (blue) and EtOH (red) injected C57BL/6 mice. **b-i)** EtOH does not affect the frequency (P=0.5514, t_{13}=0.6115) (b, f), amplitude (P=0.0774, t_{13}=1.918) (c, g), rise time (P=0.6511, t_{13}=0.4629) (d, h) or decay time (P=0.7155, t_{13}=0.3726) (e, i) of sEPSCs in the DLS (n =7 saline and n=8 EtOH from 1 mouse each). Data in **b-e** analyzed with Student's unpaired t-tests. Data represent mean ± SEM.

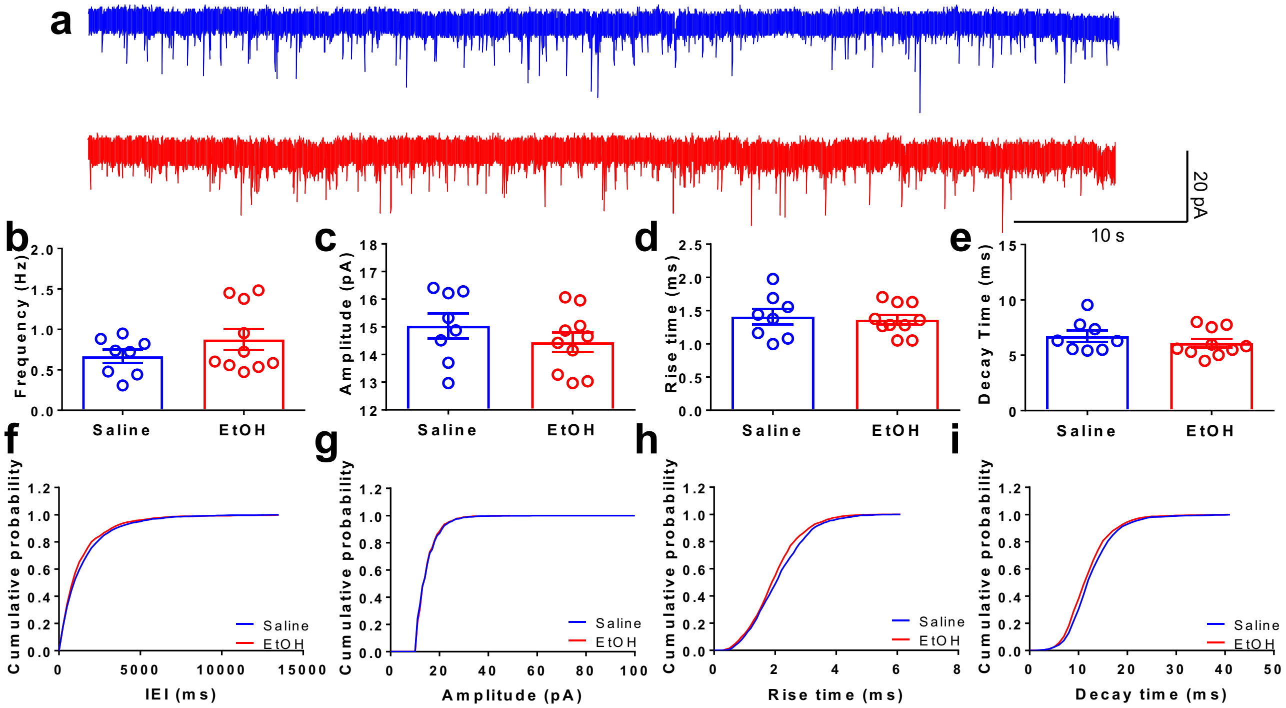

Supplementary Figure 6. A single *in vivo* EtOH exposure does not affect DLS MSN mEPSCs. a) Representative miniature excitatory postsynaptic current (mEPSC) traces of saline (blue) and EtOH (red) injected C57BL/6 mice obtained with bath inclusion of 0.5 mM TTX. **b-i)** EtOH does not affect the frequency (P=0.2238, t_{16}=1.266) (b, f), amplitude (P=0.3066, t_{16}=1.056) (c, g), rise time (P=0.7299, t_{16}=0.3514) (d, h), or decay time (P=0.3378, t_{16}=0.9882) (e, i) of mEPSCs in the DLS (n =8 saline and n=10 EtOH from 1 mouse each). Data in **b-e** analyzed with Student's unpaired t-tests. Data represent mean ± SEM.

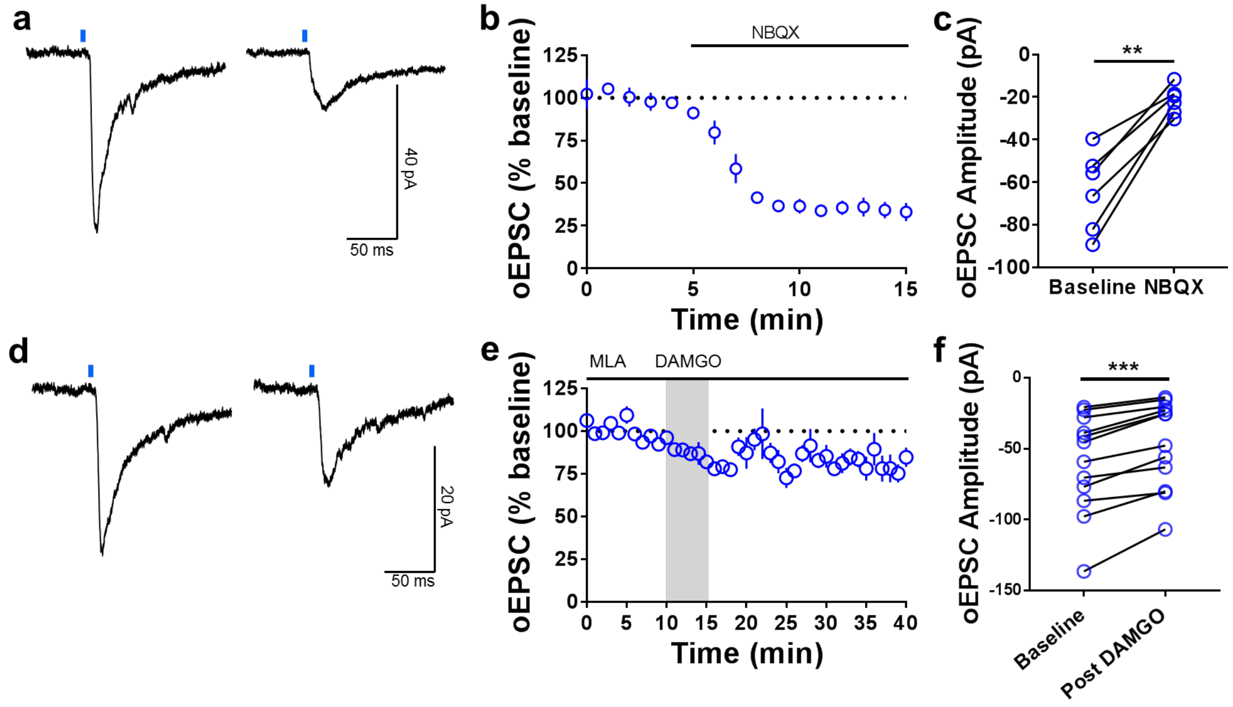

Supplementary Figure 7. MORs on CINs produce LTD. a) Representative optically evoked synaptic traces at baseline and after NBQX (5 µM, 10 min) application. **b-c)** NBQX blocks glutamatergic currents driven by CIN activation (paired t-test, P=0.012, t_5=6.54, n=6 from 1 mouse). **d)** Representative electrically evoked synaptic traces at baseline and after DAMGO (0.3 µM, 5 min) application. **e-f)** The application of MLA (100 nM during entire recording) was incapable of preventing mOP-LTD via DAMGO application (paired t-test, P=0.0001, t_{11}=6.43, n=12 from 6 mice). Data represent mean ± SEM. **P = 0.01, ***P < 0.001.

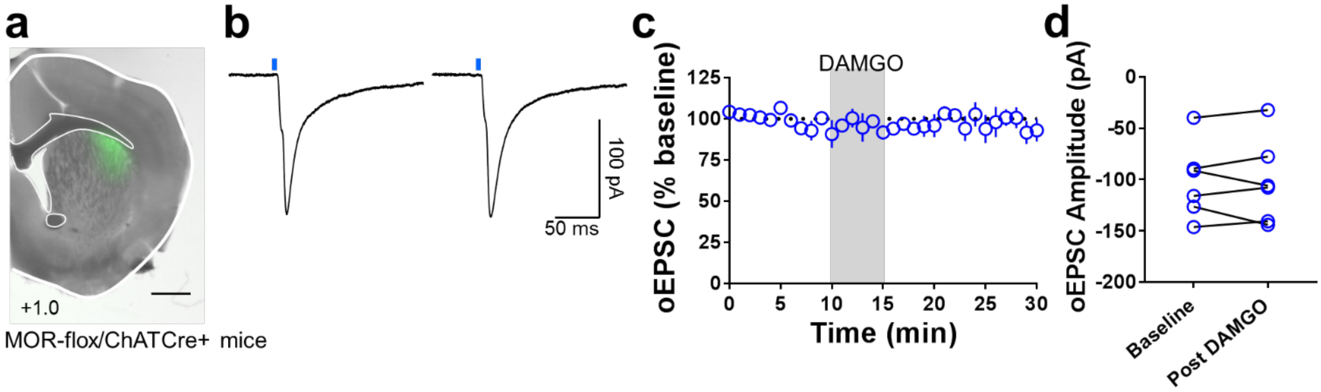

Supplementary Figure 8. MOR ablation from CINs prevents CIN-mOP-LTD in DLS. a) An AAV vector coding for ChR2 (AAV9.DIO.ChR2.YFP) was injected into DLS 14 days prior recordings in MOR-flox/ChATCre+ mice. Coronal brain slice showing the viral infection of CINs in DLS. Bar scale = 1000 µm. **b)** Representative electrically evoked synaptic traces at baseline and after DAMGO (0.3 µM, 5 min) application. **c-d)** The deletion of MOR from CINs rendered CINs incapable of producing mOP-LTD (paired t-test, P=0.988, t_5=0.015, n=6 from 2 mice). Data represent mean ± SEM.

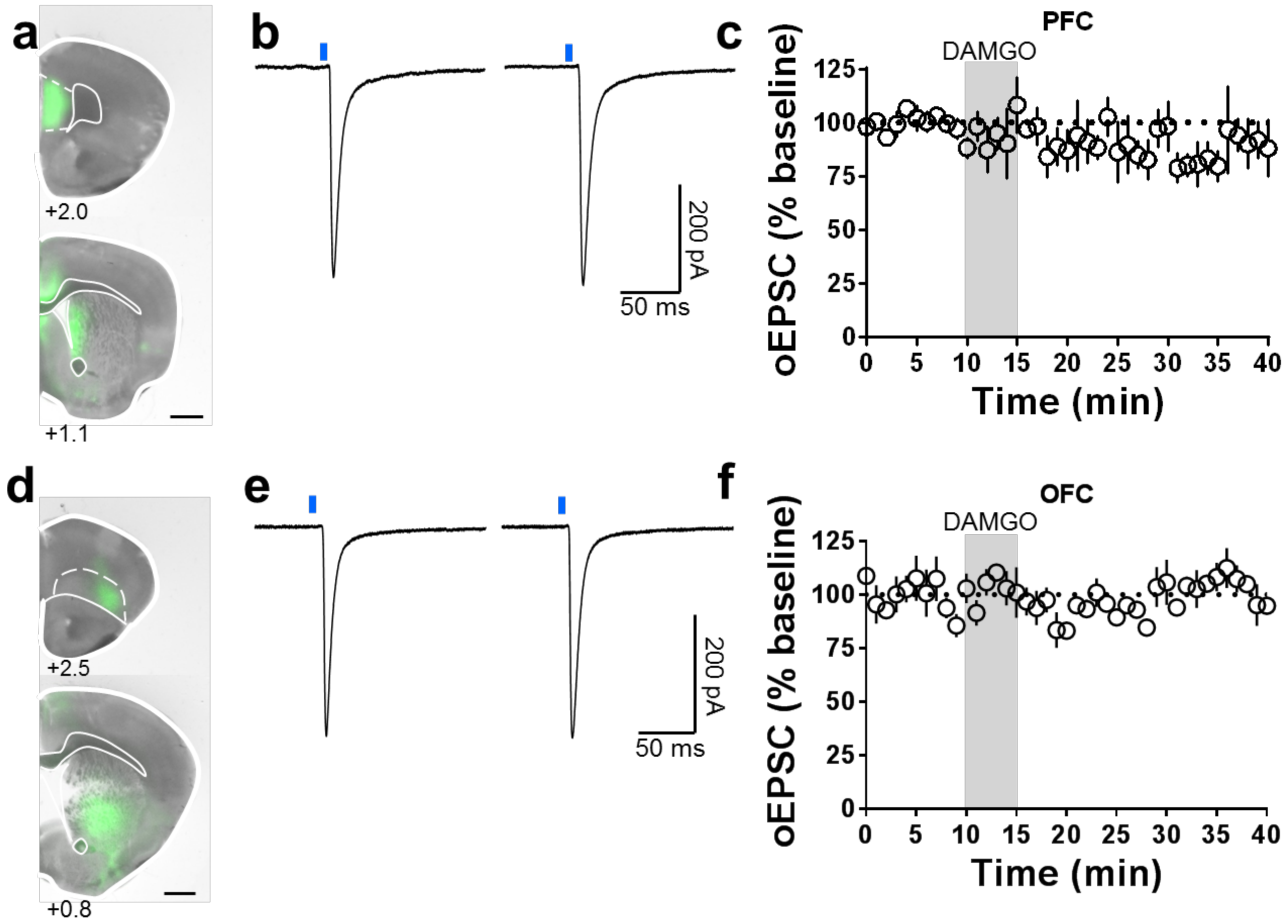

Supplementary Figure 9. mPFC and OFC are not involved in DLS mOP-LTD. a) An AAV vector coding for ChR2 (AAV.hSyn.ChR2) was injected into mPFC 14 days prior recordings in DLS. Coronal brain slice showing the infection of mPFC projections to striatum. Bar scale=1000 µm. **b)** Representative optically evoked synaptic traces recorded in DLS before and after DAMGO (0.3 µM, 5 min) application. Despite lower levels of ChR2 expression in DLS following the mPFC injection, oEPSCs were reliably obtained. **c)** No apparent effect of DAMGO was observed during mPFC input stimulation in DLS (85.1. ± 6.5%, paired t-test, P=0.145, t_3=1.958, n =4 from 2 mice). **d)** Coronal brain slice showing the AAV infection of OFC projections to striatum. Bar scale=1000 µm. **e)** Representative optically evoked synaptic traces before and after DAMGO (0.3 µM, 5 min) application. **f)** MOR activation did not produce mOP-LTD at OFC terminals in DLS (99.6 ± 4.3%, paired t-test, P=0.51, t_3=0.754, n =4 from 2 mice). Data represent mean ± SEM.

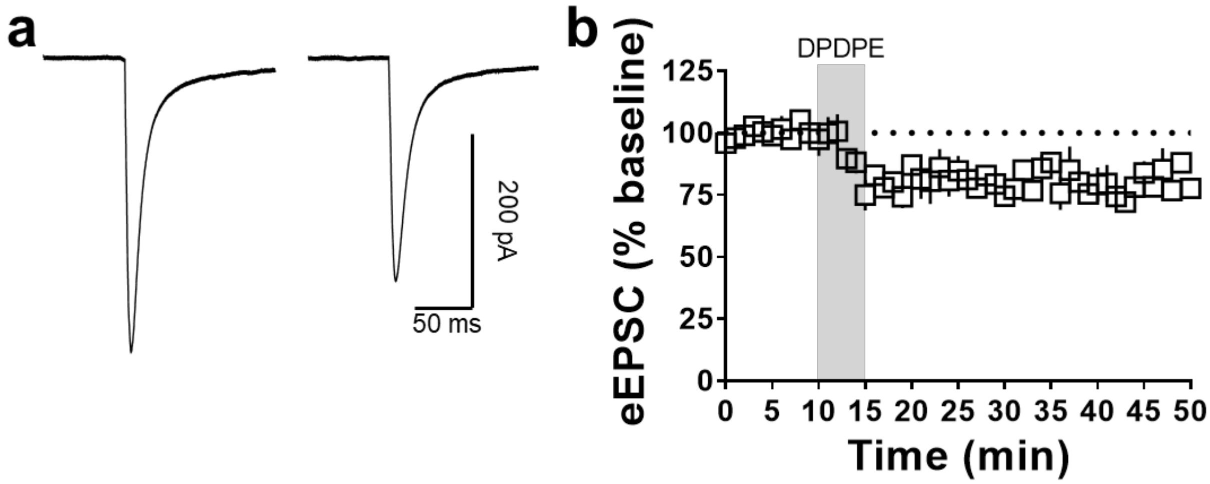

Supplementary Figure 10. DLS delta OP-LTD was not affected after MOR KO from anterior insular cortex. a) Representative electrically evoked synaptic traces before and after application of the delta opioid receptor (DOR) agonist, DPDPE (0.3 µM, 5 min), in MOR-flox mice injected in anterior insular cortex with AAV-cre vector (main text, Fig. 9a). **b)** DOR activation by DPDPE induced delta OP-LTD in the DLS of AAV-cre injected MOR-flox mice (80.0 ± 3.8%, paired t-test, $P=0.046$, $t_2=4.488$, $n=3$ from 2 mice).
